# Integration of Environmental Sustainability Principles and Climate Change Adaptation Measures in Energy Optimization at Gold Mining Operations, South Africa’s Free State Operations

**DOI:** 10.1007/s00267-025-02373-1

**Published:** 2026-02-27

**Authors:** Irene Nadunga, Richard Kwame Adom, Mulala Danny Simatele

**Affiliations:** School of Geography, Archaeology and Environmental Studies, University of Witwatersrand, Johannesburg, South Africa

**Keywords:** Environmental Sustainability Principles, Climate Change Adaptation, Energy Optimization, Gold Mining

## Abstract

In light of the unsustainable energy consumption and significant greenhouse gas emissions threatening South Africa’s gold mining sector, this study examined how environmental sustainability principles and climate change adaptation measures are integrated to enhance resilience and energy efficiency. Guided by three research questions; identifying climate change impacts on energy use, assessing sustainability practices implemented by mining companies, and exploring how a conceptual framework can guide integration; the study employed a mixed-methods case study approach using purposive and snowball sampling of 30 participants across ten Witwatersrand Basin operations, complemented by documentary reviews, site observations, and quantitative climate and energy data. Findings revealed that mean annual temperatures in the Free State Province are projected to rise by +2.3 °C, with very hot days (>35 °C) nearly doubling, intensifying thermal stress and driving cooling demand; yet, electricity consumption declined from 1231 GWh in 2020 to 1071 GWh in 2023 due to targeted efficiency programmes. Interviews confirmed strong awareness among sustainability officers and mining experts, with 100% reporting adoption of energy-saving initiatives such as optimized refrigeration, advanced ventilation systems, and seasonal cooling controls, while 77–83% emphasized energy efficiency as both a sustainability principle and adaptation strategy. Overall, the study demonstrates that climate change is reshaping energy consumption patterns, but proactive integration of energy efficiency and renewable energy projects can simultaneously reduce costs, lower emissions, and strengthen resilience. These findings imply that embedding sustainability into adaptation frameworks is essential for ensuring the long-term viability of gold mining operations and aligning industry practices with national and global sustainability goals.

## Introduction

Sustainable development involves three dimensions: economic growth, social development, and environmental sustainability that are interlinked (World Bank, 2016). However, Bhattacharya (2015b) notes that achieving balance among these dimensions remains a challenge to global institutions. In December 2015, 125 countries, including South Africa, signed the Paris Agreement on climate change to reduce greenhouse gas (GHG) emissions by 80% by 2050 (Wei et al. 2021). Subsequently, in September 2015 at the United Nations Sustainable Development Summit, nations including South Africa adopted the 2030 Agenda for Sustainable Development, with 17 Sustainable Development Goals (SDGs) at its core (World Bank 2016). The international community’s adoption of the SDGs marked recognition that economic growth, poverty reduction, and environmental sustainability are intricately linked (World Bank 2016). However, achieving sustainable development remains a challenge (World Bank 2016), particularly regarding clean energy production and reducing GHG emissions from fossil fuels (United Nations, 2017). These challenges are pronounced in Africa, given the continent’s vulnerability to climate extremes (United Nations Economic and Social Council [UNESC], 2015; Centre for International Governance Innovation [CIGI], 2009). Sub-Saharan Africa shows uneven economic growth (Peschka, 2014), despite being one of the fastest-growing regions globally (International Finance Corporation [IFC] 2014). Climate change is a key factor contributing to this uneven growth (Connolly-Boutin and Smit 2016).

South Africa is not immune to climate impacts, experiencing decreased precipitation, heat waves, and recurrent droughts (Connolly-Boutin and Smit 2016). These variabilities exacerbate energy challenges due to rising demand and limited infrastructure (Connolly-Boutin and Smit 2016). Mining contributes significantly to South Africa’s economy (Minerals Council South Africa 2022; IFC 2014). In 2022, the sector employed 469,353 people and contributed R483.3 billion to Gross Domestic Product (Minerals Council South Africa 2022). Despite this, mining is highly energy-intensive, relying on Eskom’s coal-fired power stations (Minerals Council South Africa, 2022; Müller, 2018b). Electricity costs have risen over 500% in the past decade (Minerals Council South Africa 2022). Müller (2018b) further notes that energy costs increased operating expenses by 10–20%, with electricity prices projected to rise from R0.70/kWh to R1.40/kWh by 2030.

Scholars such as Bartos (2007), Humphreys (2019), and Azadi et al. (2020) argue that climate change drives high mining costs. Rising global temperatures increase cooling and ventilation demands (Ebi et al. 2021), while extreme weather disrupts operations and damages infrastructure. Declining ore grades require more energy-intensive processing (Firoozi et al. 2024). Heat stress reduces worker productivity and equipment efficiency (Department of Economic, Small Business Development, Tourism and Environmental Affairs [DESTEA] 2024). Stricter carbon regulations and energy transitions necessitate costly upgrades (Santaro et al. 2021). Collectively, these factors make climate change a central contributor to escalating mining costs (Shemer et al. 2023).

In light of the increased and unsustainable consumption of energy and significant emissions GHG threatening the mining operations in South Africa, very limited literature exists to address this phenomenon. Considering these gaps this paper explored how environmental sustainability principles and climate change adaptation measures are integrated to enhance the resilience and energy efficiency of gold mining operations in South Africa, under the following research questions: (i) What are the key climate change impacts affecting energy consumption in South African gold mining operations?, (ii) What environmental sustainability practices are currently being implemented by gold mining companies to reduce energy use and emissions?, and (iii) How can a conceptual framework guide the integration of sustainability and adaptation strategies in the mining industry? The remainder of the paper is divided into five sections. The literature review is covered in section two of this paper, relating to energy consumption, climate change adaptation and sustainability strategies in the mining industry. The third section explores the study’s methodology, while section four outlines the empirical evidence and analysis of the research. Finally, the fifth and sixth sections of this paper, present the study’s discussions, conclusion and recommendations.

## Literature Review

### The Link Energy Consumption and Environmental Degradation in the Mining Sector

Mining’s reliance on fossil fuels globally and in South Africa drives greenhouse gas emissions, air pollution, and climate change (Enemou et al. 2023). Large-scale projects, such as Australia’s coal sector and China’s rare earth mining, add significant carbon and toxic waste (Agboola et al. 2020). In South Africa, mining consumes about 15% of electricity mostly coal-based, intensifying environmental problems in Mpumalanga’s heavily polluting coal plants (Tladi et al. 2024). Deep-level gold mining in Witwatersrand worsens acid mine drainage, contaminating rivers like the Vaal (Durand 2012), while open-pit mining accelerates deforestation and biodiversity loss in places such as the Amazon and Phalaborwa copper mine (Espejo et al. 2018). Overall, the sector’s high energy demands degrade ecosystems and create long-term socio-economic challenges, including pollution, water scarcity, and community displacement (Leyton-Flor et al. 2024).

### Climate Change Adaptation and Sustainability in the Mining Industry

Existing studies affirm that climate change adaptation measures are essential for achieving the SDGs by fostering climate-resilient development (Berbés-Blázquez, 2017; Rasul and Sharma 2016). Scholars such as Uitto and Berg (2017) and Liu et al. (2017) highlight the interdependence between adaptation and sustainable development, stressing the need for integrated models, innovative methodologies, and decision-support tools. Adaptation is defined as reducing adverse climate impacts on human and natural systems, requiring transformation across social, economic, and environmental spheres. Bouhia (2018) underscores the importance of integrated responses for resilience, though Kolk and Pinkse (2011) caution that synergies may falter in complex socio-economic contexts. Despite investment, adaptation implementation remains limited due to behavioural, institutional, and governance barriers (Leiter 2017; Wise et al. 2014). Nelson and Schuchard (2011) further note that mining-sector adaptation often prioritizes engineering solutions over broader sustainability considerations, neglecting site-specific social risks and opportunities.

In light of these findings, there is a pressing need for mining and other climate-sensitive industries to develop robust adaptation policies, and to integrate adaptation strategies more deeply into planning and operational processes.

### Sustainability strategies, Climate change adaptation and Energy use in Mining

The mining industry plays a dual role in sustainable development, offering economic contributions while posing ecological challenges. While some scholars highlight its importance in cost-effective mineral extraction for global sustainability (Soler and Marcé 2018; Odell et al. 2018), others stress that environmental impacts undermine this potential (Shen et al. 2015). As a result, mining companies are urged to adopt responsible practices, including green supply chain management and decision-making frameworks that align operations with sustainability goals (Shen et al. 2015; Govindan 2015). Climate change further complicates this role, intersecting with multiple domains critical to development planning (Connolly-Boutin and Smit 2016; Hens 2010), though synergies between climate action and development are not always guaranteed (Kolk and Pinkse 2011). Scholars emphasize the need for comprehensive climate management systems, adaptation pathways, and proactive risk strategies (Wise et al. 2014; Nelson and Schuchard 2011; Chersich and Wright 2019; Carbon Disclosure Project [CDP] 2017). Energy efficiency emerges as a key leverage point, linking mitigation with sustainable development (Beg et al. 2002; Davidson et al. 2003; Rugină and Badea 2016). In Africa, mining companies increasingly adopt renewable energy to meet the triple goals of mitigation, adaptation, and sustainability, reflecting a broader shift toward proactive climate adaptation (Elum and Momodu 2017a; Ley 2017a; Farrant 2016; Perdeaux 2017).

## Materials and methodology

### Study Location

This study was conducted at gold mining operations in the Witwatersrand Basin, primarily within South Africa’s Free State province. Mining remains central to the provincial economy, contributing 4.9% to national GDP between 2019 and 2022. Gold dominates mineral production, with Free State Consolidated Goldfields as the country’s largest complex. The province hosts 12 gold mines, producing about 30% of South Africa’s total output (DESTEA 2024).

The site was selected for its geographical setting and broader institutional, environmental, and socio-economic context. The Witwatersrand Basin extends 250 miles from Johannesburg to Welkom, with gold-bearing reefs along its northern and western margins (Tucker et al. 2016). Mining here spurred the growth of cities and towns, shaping South Africa’s economy (Tucker et al. 2016). However, mining also heightens community vulnerability to climate change impacts such as rising temperatures and water shortages (PAGE 2016; Peschka 2014), while ecosystems have been degraded (Dye and Weiersbye 2010).

**Fig. 1:**
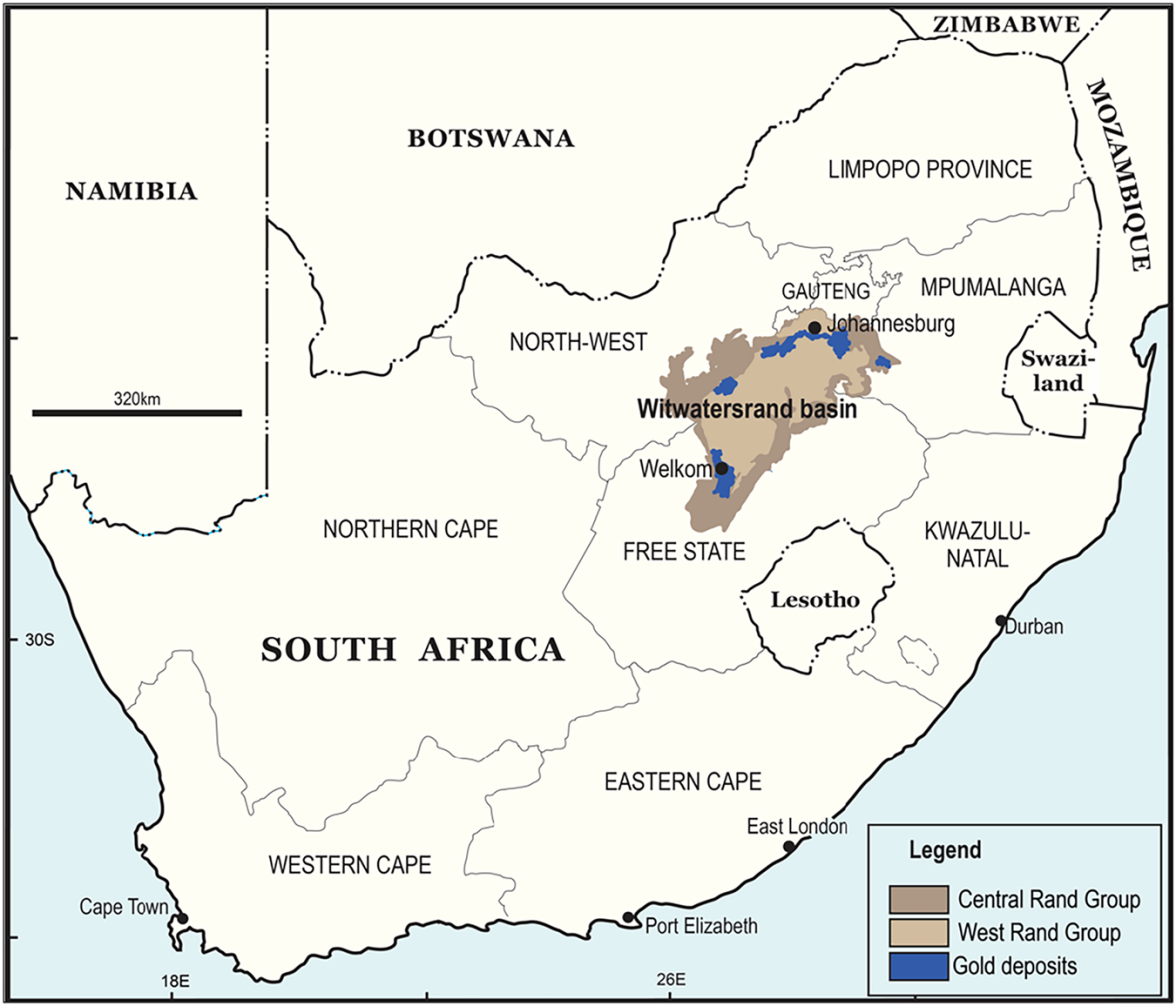
Location of the Witwatersrand Basin in South Africa with Free State Province Consolidated Goldfields as the country’s largest complex (Source: Cartographic Unit, University of the Witwatersrand’s Department of Geography and Environmental Studies, 2022)

Environmental pressures have intensified due to mine abandonment during economic downturns and irresponsible practices (IFC 2014; Nelson and Schuchard 2011). These challenges underscore the need for sustainable mining, especially in sensitive regions (Kogel et al. 2014). This study focuses on selected operations within the Witwatersrand Basin, which remain unnamed to preserve anonymity as required by ethics clearance. Participants are similarly coded to ensure confidentiality. Figure 1 shows the basin’s location in northeastern South Africa.

### Climate Vulnerability of the Mining Sector

**Fig. 2:**
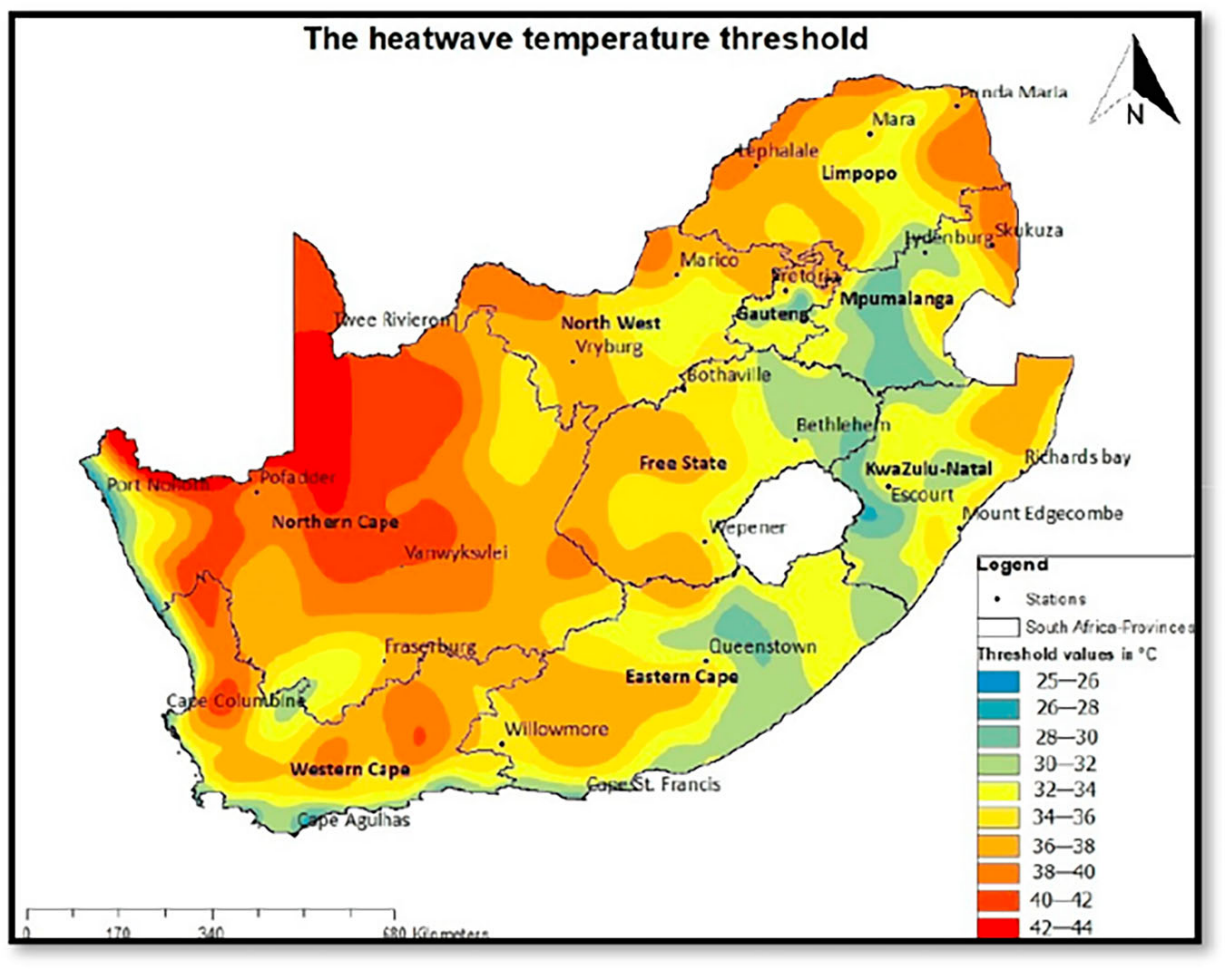
Heatwave Temperature Threshold in South Africa with Free State consolidated goldfields complex temperature threshold ranging between 32 °C and 38 °C (Source: Fazel-Rastgar and Sivakumar 2024)

The mining sector’s vulnerability to climate change is heightened by its reliance on water for operations, particularly in gold mining. Rising temperatures also pose health risks to mine workers, increasing the incidence of heat-related illnesses. In the Free State province, both minimum and maximum temperatures have shown consistent increases, with climate models projecting further rises (DESTEA 2024). Heatwaves are becoming more frequent and intense, with thresholds ranging between 32 °C and 38 °C (see Fig. 2). The South African Weather Service defines a heatwave as a period when maximum temperatures exceed the average maximum of the hottest month by at least 5 °C for three or more consecutive days (South African Weather Service n.d.). Very hot days are classified as those exceeding 35 °C (DESTEA 2024).

South Africa experienced a severe heatwave in January 2023, with maximum temperatures ranging from 33 °C to 36 °C between January 10 and 18, peaking at 36.15 °C. In Bloemfontein, where the heatwave threshold is 35 °C, daily maximums between January 11 and 15 ranged from 34 °C to 36 °C, specifically recording 35 °C, 35 °C, 34 °C, 36 °C, and 35 °C (Fazel-Rastgar and Sivakumar 2024).

### Sampling techniques and sample population

This study employed a non-probability sampling strategy, commonly used in case study research. Gold mining operations in South Africa with varying sustainability initiatives were purposefully selected, along with employees whose roles involved climate change, sustainable development, and energy management. This approach ensured alignment with the study’s objectives and targeted participants with relevant expertise.

Ten gold mining operations within the Witwatersrand Basin were chosen for convenience and accessibility. With ethics clearance, Human Resources departments assisted in identifying 34 employees in middle and senior management positions across the Environment, Sustainable Development, and Engineering sections. These participants were assumed to have substantive knowledge of sustainability-related processes within their operations. Purposeful sampling was advantageous in capturing specialized insights but carried risks of selection bias, such as overrepresentation of sustainability-focused employees or overly positive reporting. To mitigate this, triangulation with documentary evidence complemented interview data (Taherdoost 2016; Creswell 2009).

Of the 34 targeted employees, 21 were available to participate. To expand the sample, snowball sampling was adopted, whereby participants recommended colleagues and contractors working in the same mining disciplines. This yielded nine additional participants, whose eligibility was confirmed during interviews and questionnaires. Snowball sampling allowed access to hidden or specialized participants and enriched the dataset (Taherdoost 2016; Creswell 2009).

Together, these techniques complemented each other by combining deliberate selection for relevance with network-based expansion, thereby enhancing both the depth and breadth of insights into sustainability practices within the gold mining operations. In total, 43 participants were identified; 34 through purposive sampling and nine through snowball sampling. Ultimately, 30 agreed to participate, a number considered sufficient for developing a well-saturated theory (Creswell et al. 2007).

### Data Collection Methods

This study employed multiple data and in-depth data collection techniques using a mixed-methods approach. A mixed method approach of quantitative and qualitative was applied in sourcing data at different times, locations, and contexts. Quantitative data on energy consumption and climate trends (temperature and heatwaves) from 2020 to 2023 were obtained from mining company operational reports and external weather providers. Qualitative data were gathered through case studies, company sustainability reports, and structured and unstructured questionnaires, complemented by interviews with sustainability officers and mining experts. Questions were designed to address the study’s three themes: climate change, environmental sustainability, and energy management. Interviews and questionnaires were self-administered via telephone, face-to-face, or email. Open-ended responses were coded and quantified to create a comprehensive dataset, with frequency counts used to analyze recurring themes (Driscoll 2007).

A rapid appraisal approach was also applied, reviewing mining company documentation identified during interviews and questionnaires. Only publicly available documents published between 2020 and 2023 were included (Cardno 2018). In addition, site observations were conducted using field checklists developed from interviews, questionnaires, and document reviews. These checklists provided ground-truth validation during site visits.

Finally, a synthesis approach integrated findings from both quantitative and qualitative analyses to develop a conceptual framework linking sustainability practices with climate adaptation measures.

### Data Analysis

Quantitative data comprising energy consumption and temperature/heatwave trends were obtained from secondary sources and organized using Microsoft Excel. Historical and projected climate data, including temperature rise, very hot days, and heatwaves, were identified and analyzed for the periods 2020–2039 and 2020–2050. However, actual recorded heatwave events for specific study sites in the Free State Province between 2020 and 2023 were not available. Baseline and projected climate variations, particularly the frequency of heatwaves and extreme temperatures, are presented graphically to illustrate trends over time and highlight changes across different years within the Free State Province. In parallel, yearly energy consumption trends for gold mining operations in the province were analyzed for the period 2020 to 2023, aligning with the timeframe of temperature and heatwave projections. Excel’s statistical tools were used for data plotting and analysis.

Qualitative data, including insights from sustainability officers, mining experts, and excerpts from document reviews, were analyzed using content and document analysis methods. This data was systematically organized, grouped into themes and categories, and then quantified to complement the separately collected quantitative data (Cardno 2018). Through this approach, the study drew inferences by identifying recurring themes, keywords, and narratives aligned with the core research themes (climate change, environmental sustainability, and energy management). Thematic analysis was employed to extract common climate concerns and perceptions of energy use, helping to identify overarching trends and patterns (Stemler and Bebell 1999). All qualitative data was manually constructed from interview and questionnaire responses into qualitative codes and organized in Microsoft Excel, where quotes and narratives were contextualized by linking them to specific events and counted for frequency. The frequencies were then converted into percentages through descriptive statistical analysis to determine the frequency distribution of identified themes, expressed as numbers or percentages (Manikandan 2011). Percentages were calculated using Excel by dividing the frequency of responses within a given category by the total number of participants and multiplying by 100% (Driscoll 2007). The summarized data was presented in various formats, including tables and graphs, to visually represent key findings and provide a clearer understanding of the results (Williams 2007; Thomas and Brubaker 2007).

## Empirical Evidence and Analysis

The findings from the data collection and analysis are structured around the research questions relating to how environmental sustainability principles and climate change adaptation measures are integrated to enhance the resilience and energy efficiency of gold mining operations in South Africa. In view of this, during the document review, interview and questionnaire processes, a number of discussions were held with the identified research participants working at the gold mining operations in effort to further understand what is presented in literature and what is experienced at the gold mining operations.

### Effects of Climate change on Energy use in Gold Mining

One of the key thematic focuses of this study was to assess the impact of climate change on energy consumption in South African gold mining operations. Content and document analysis of gold mining company sustainability reports revealed that climate change and extreme weather susceptibility are recognized as material risk to the business. As a result, its impacts are explicitly addressed in company policies and strategic planning. Notably, excerpts from the 2022 Environmental Social and Governance (ESG) mining company report highlight that excessive heat is considered a significant climate-related risk with the potential to substantially affect operations. Excerpts from the sustainability reports are noted as:

*“Material climate-related risks, which could have substantive financial impacts, include safety (due to excessive heat….)”* (ESG company report, 2022).

*“Climate change is the most serious environmental risk confronting our business. We are susceptible to extreme weather events such as increasing temperatures that could affect underground ambient temperatures….”* (ESG company report, 2022)

**Fig. 3:**
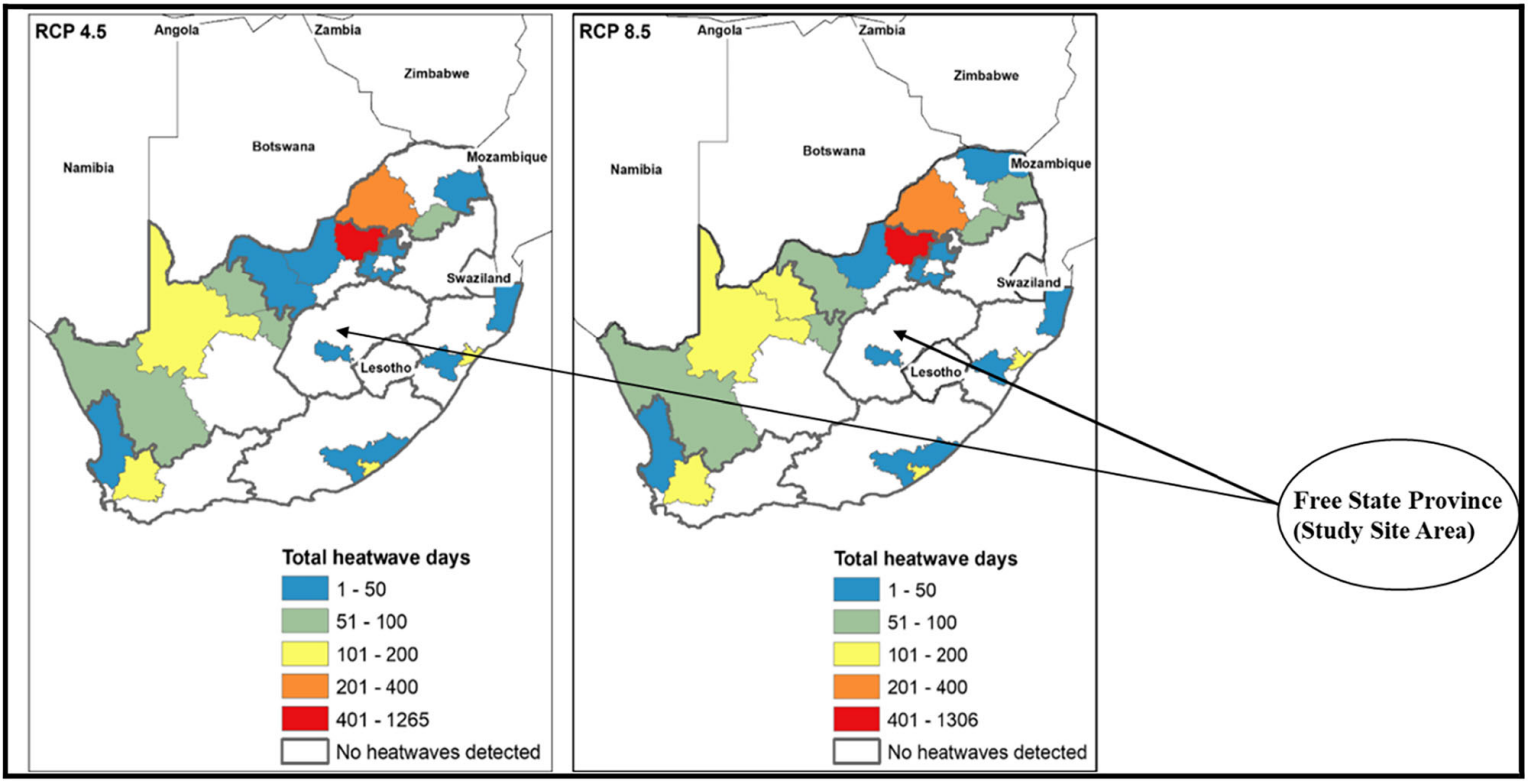
Simulated heatwaves in Free State province of South Africa during the period 2020 – 2039 (19 years) under RCP scenarios 4.5 and 8.5 (Source: Kapwata et al. 2022)

**Fig. 4:**
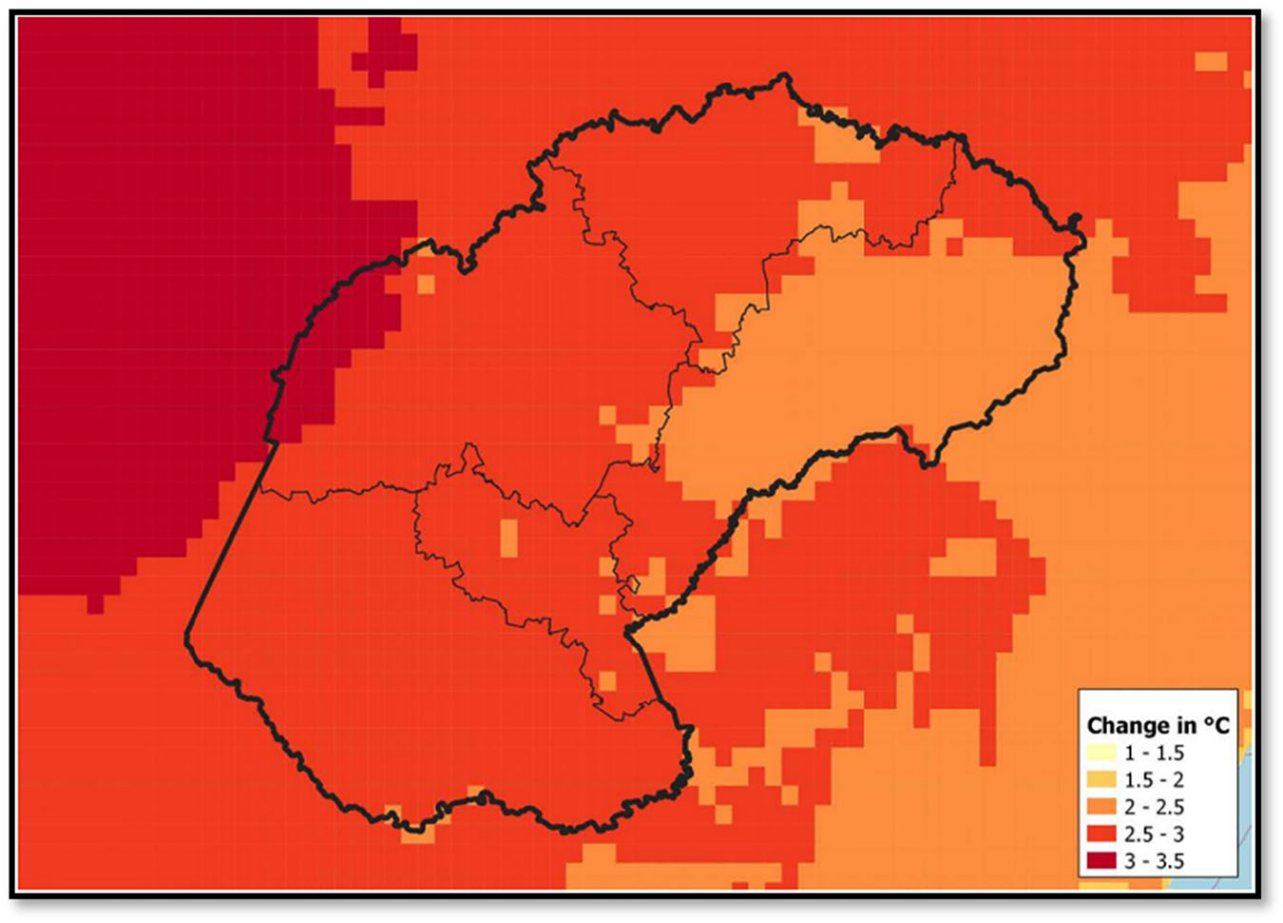
Projected changes in Average Annual Maximum Temperature (Tmax) in the Free State Province for the period 2021 to 2050 (Source: DESTEA, 2024)

Findings from the content analysis of historical (baseline) and projected climatic data of the study site is presented in Table 1: Mean annual temperature, historically ~21 °C, projected to rise by +2.3 °C, indicating significant warming across the province. Average annual maximum temperature (Tmax), baseline of ~29.5 °C, expected to increase by +2.3 °C, intensifying seasonal heat extremes. Very hot days (>35 °C): Baseline of 88 days per year, projected to increase by +54 days, nearly doubling the frequency of extreme heat. Under the Representative Concentration Pathway (RCP) 8.5 scenario, these increases relative to the historical baseline are illustrated in Fig. 3 for the period of 2020–2039 and Fig. 4 for the period 2021 to 2050.

**Table 1:** Showing projected changes in climate variables 2020 – 2050 for the Free State province of South Africa (Baseline climatology: 1970–2015; Sources: DESTEA, 2024; Kapwata et al. 2022; Climate-Data.org, 2025)

Climate Variable	Baseline (1970 – 2015)	Change under the RCP8.5 Scenario (2020 – 2050)	Notes
Mean annual temperature	~21.1 °C (Climate-Data.org, 2025)	+2.3 °C	Province-wide climatological mean
Average annual maximum temperature (Tmax)	~29.5 °C (DESTEA, 2024)	+2.3 ^o^C	Clarified as Tmax, not mean
Very hot days (>35 °C)	88 days (DESTEA, 2024)	+54.15 days	Frequency of extreme heat
Heat wave events	13.9 events (Kapwata et al. 2022)	+17.5 events	Defined as ≥3 consecutive very hot days

Fig. 3 presents future projections indicating increased frequency of heatwaves under both RCP4.5 and RCP8.5. In some subregions, the total number of heatwave days could reach up to 50 during 2020–2039 (Kapwata et al. 2022).

Figure 4 illustrates the projected changes in average annual maximum temperatures (Tmax) in the Free State Province for the period 2021 to 2050, relative to the historical baseline. Rising Tmax values are expected to significantly increase both the frequency of heatwave events and the number of extremely hot days. Compared to the historical baseline ( ~ 29.5 °C Tmax), projections show +2.3 °C increases, consistent with climate change impacts.

**Fig. 5:**
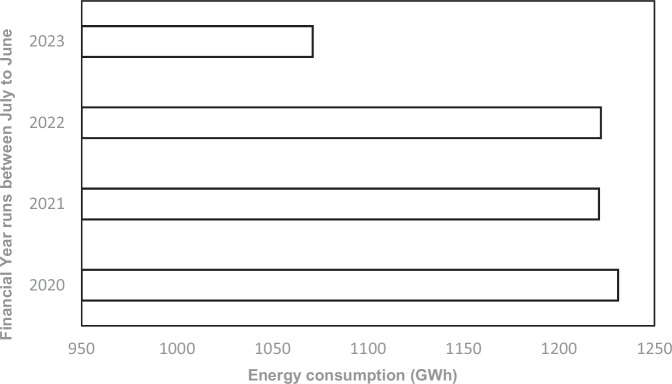
Total Energy Consumption for the Gold Mining Operations in the Free State province between 2020 and 2023 (Source: Gold mining company operational reports, 2020–2023)

Gold mining operations in the Free State Province are predominantly deep underground mines, which are typically more energy-intensive than surface mining operations. According to the 2023 ESG report from the gold mining company, energy consumption remains a major financial and environmental concern, as mining and extraction processes demand substantial energy inputs, significantly influencing operating costs. Figure 5 shows a gradual decline in total energy consumption by gold mining operations in the province between 2020 and 2023, with a particularly notable reduction in the 2023 financial year, where electricity consumption decreased by 3.6%. This reduction is largely attributed to the successful implementation of energy efficiency and management programmes. Excerpt from the sustainability reports is noted as:

*“We reduced our electricity intensity by 46% over the past 10 years with our commitments to optimise energy efficiency and climate change mitigation.” (ESG company report,* 2022).

Projected increases in maximum temperatures, very hot days, and heatwave events (Fig. 4) highlight the growing thermal stress on underground gold mines, which would normally drive higher electricity demand for cooling, ventilation, and compressed air systems. However, despite these climatic pressures, energy consumption declined from 1231 GWh in 2020 to 1071 GWh in 2023 (Fig. 5), reflecting the success of targeted efficiency programmes that offset the otherwise rising demand. This decline demonstrates that proactive upgrades in cooling and ventilation systems were a direct response to projected heatwave risks, with the observed reductions serving as evidence of effective adaptation rather than diminished climatic pressure.

According to the 2023 ESG company report, over 200 energy efficiency initiatives have been implemented across the operations. Notably, some of these initiatives aim to manage underground temperatures for employees exposed to excessive heat and to regulate ventilation to prevent temperatures from exceeding safe working limits. Excerpts from the sustainability reports are noted as:

*“We are aware of the need for more efficient energy consumption. Our energy efficiency initiatives focus on mine cooling, compressed air, and ventilation.”* (ESG company report, 2023)

*“…. implemented optimised compressor air valve control and synchronised it with the Missing Person Locator (MPL) system. Fan optimisation providing for seasonal ventilation control based on ventilation requirements and optimising on fan running combinations according to downscaling management plan; and optimised improved refrigeration.”* (ESG company report, 2023)

**Fig. 6:**
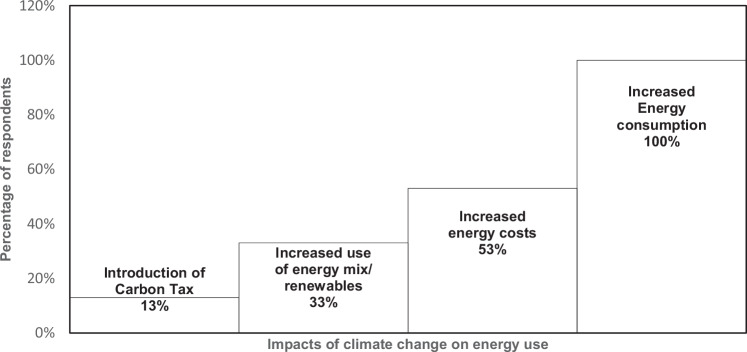
Perceptions of how climate change impacts on energy consumption in the gold mining operations (Source: Field based data of 2020 and 2021)

Findings from interviews and questionnaires, presented in Fig. 6 indicate a strong awareness among the research participants (sustainability officials and mining experts) that climate change is impacting energy consumption in gold mining operations. All research participants (100%) acknowledged that rising temperatures linked to climate change have led to increased energy use, primarily to enhance cooling efforts in underground mining environments. Additionally, 33% of participants highlighted the growing need for gold mining operations to diversify their energy mix by incorporating renewable energy sources, as an alternative to exclusive reliance on fossil fuels, which are significant contributors to GHG emissions. This perspective aligns with the gold mining company’s Task Force on Climate-related Financial Disclosures [TCFD] report (2021), which emphasizes a shift toward less energy-intensive mining methods. The report also notes the company’s recognition of opportunities to improve operational energy efficiency as a means to reduce both energy costs and associated GHG emissions.

### Environmental sustainability practices used to conserve Energy in Gold Mining

This study also aimed to evaluate the environmental sustainability practices in South African gold mining, with a specific focus on energy use. Through content and document analysis of the gold mining company sustainability reports, it was found that several renewable energy projects were being progressively incorporated into the energy mix. These projects included solar PV, wheeling wind energy, hydropower, and energy efficiency initiatives. The primary goals of these efforts were to reduce electricity consumption and lessen dependence on Eskom’s fossil fuel-based power. Excerpts from the sustainability reports are noted as:

*“We are exploring a number of self-generation renewable energy options, including solar projects.”* (ESG company report, 2021)

*“Increase in renewable energy consumption as a percentage of total energy mix.”* (ESG company report 2023)

Additional findings from the content analysis of responses gathered during interviews with sustainability officials and mining experts are presented in Table 2.

**Table 2:** Perceptions of how gold mining operations conserve energy resource ‘*N* = 30’

Participants’ Responses	Frequency	Percentage of Respondents
Implemented energy saving initiatives and project	30	100%
Monitoring and maintenance for equipment efficiencies (Optimisation)	21	70%
Use of renewable energy	14	47%
Awareness raising	12	40%
Monitoring and reporting on environmental performance	11	37%
Implemented policies and procedures	9	30%
Conducting audits	9	30%
Capital funding/investment	9	30%
*Others	6	20%

**Source:** Field based data (2020 and 2021)

*Others, represents all the responses received with a frequency less than 5

All research participants (100%) agreed that gold mining operations are conserving energy through the implementation of various energy-saving initiatives. These initiatives include optimizing refrigeration units, improving ventilation systems by installing variable speed drives and inlet guide vanes on main fans, optimizing seasonal cooling controls, and reducing electricity consumption during peak periods. Interestingly, a minority of participants (20%), categorized as “Others”, noted that while these initiatives are being implemented, they are not always driven by a deliberate intent to conserve energy. Instead, they suggested that in some cases, the primary motivation is to reduce operational costs. This was captured by some of the key participants stating that:

*“…Energy savings are primarily motivated by reducing operational costs due to high electricity prices.”* (Pers. com, 2020a).

This is further supported by statements in the sustainability reports, which highlight that the primary objective of the renewable energy programs implemented by gold mining operations is to reduce costs associated with purchasing electricity from Eskom.

*“Additional challenges included Eskom’s load curtailment (frequent requests to reduce power consumption) and tariff increases, which heightened the urgency to implement renewable energy projects.”* (ESG company report, 2023)

*“Incentives driving our renewable energy programme include cost savings through purchasing less grid electricity from our constrained supplier, Eskom.”* (ESG company report, 2023)

### Integration of Sustainability and Adaptation Strategies in Gold Mining

To support the development of a conceptual framework that integrates sustainability principles with climate change adaptation measures, this study examined the relationship between environmental sustainability principles and the adaptation strategies employed by gold mining operations. The findings, drawn from content and document analysis as well as interviews and questionnaire responses, are presented below.

As shown in Table 3, the majority of participants (40%) indicated that gold mining operations are implementing energy efficiency measures as a key environmental sustainability principle, particularly in relation to the sustainable use of energy resources to address climate change impacts. Additionally, 10% of participants supported this view, emphasizing that by using resources sustainably, gold mining operations are actively applying environmental sustainability principles to mitigate climate change effects. Similarly, 37% of participants highlighted that these gold mining operations are working to reduce their carbon footprint by enhancing energy efficiency and aligning with broader sustainability goals.

**Table 3:** Sustainability principles implemented to address the impacts of climate change ‘*N* = 30’

Participants’ Responses	Frequency	Percentage of Respondents
Implement energy efficiency measures	12	40%
Reduced carbon footprint	11	37%
Legal compliance	10	33%
Implementation of projects	9	30%
Paying Carbon Tax	8	27%
Providing capital funding	8	27%
Associated operational cost reduction	7	23%
Not applicable	5	17%
Sustainable use of resources	3	10%
Community Empowerment	2	7%
Carbon Disclosure project (CDP)	2	7%
Availability of resources	1	3%

**Source:** Field based data (2020 and 2021)

To further explore the connection between environmental sustainability principles and energy optimization, participants were asked how gold mining operations are applying these principles to improve energy consumption. Interestingly, the responses summarized in Table 4 closely mirror those presented in Table 3, which relate to the implementation of sustainability principles to address the impacts of climate change.

**Table 4:** Sustainability principles applied to achieve energy optimization ‘*N* = 30’

Participant’s Responses	Frequency	Percentage of Respondents
Implementing energy efficiency- saving measures (Optimisation)	25	83%
Reduced carbon footprint	11	37%
Capital funding	8	27%
Conducting audits	8	27%
Mine departmental structuring	4	13%
Alternative Energy sources	4	13%
Not applicable	3	10%
Stakeholder consultations	1	3%
Training	1	3%

**Source:** Field based data (2020 and 2021)

The majority of participants (83%) in Table 4 believe that gold mining operations are applying the principle of sustainable resource consumption by implementing energy efficiency and conservation measures. Supporting this view, a smaller group of participants (13%) further noted that these operations are adopting sustainability principles through the use of alternative energy sources. This approach aims to optimize energy consumption and reduce dependence on fossil fuel-based energy, which in turn helps lower the mine’s carbon footprint, as highlighted by 37% of participants. One participant remarked that:

*“…The gold mining operations are moving towards the increased use of renewable energy where appropriate”* (Pers. com, 2020b).

Another participant provided an example of a renewable energy project being implemented at one of the gold mining operations:

*“…Through the use of renewable energy e.g., the Bio Gas Project”* (Pers. com, 2020c).

To further explore the link between climate change adaptation measures and energy optimization, participants were asked how gold mining operations are implementing adaptation strategies to achieve energy optimization. The responses, presented in Table 5, are notably similar to those in Tables 3 and 4.

**Table 5:** Climate change adaptation measures implemented in an effort to conserve energy ‘*N* = 30’

Participant’s Responses	Frequency	Percentage of Respondents
Implementing energy efficiency measures and projects	23	77%
GHG reduction initiatives	16	53%
Not applicable	8	27%
Conducting feasibility studies	8	27%
Conducting audits	8	27%
Strategic Risk Management	2	7%
Use of Renewable/ energy mix	2	7%
No climate change adaptation measures	2	7%
Implementing Company policies and procedures	1	3%
Climate change projection/ Scenario Planning	1	3%

**Source:** Field based data (2020 and 2021)

Table 5 shows that the majority of participants (77%) believe that gold mining operations are implementing energy efficiency measures and projects designed as climate change adaptation strategies. These measures aim to optimize energy consumption while addressing the impacts of climate change. Some participants cited examples such as the Bioenergy project, where energy crops are cultivated to promote carbon sequestration and are used as biomass for electricity generation, resulting in reduced GHG emissions. Similarly, 53% of participants indicated that GHG reduction initiatives are being adopted by gold mining operations as part of their climate change adaptation strategies to optimize energy use and mitigate climate change effects. Additionally, 7% of participants noted that renewable energy is being integrated into the energy mix, serving both as a climate change adaptation measure and an energy optimization initiative.

These findings are further supported by statements in the sustainability reports, which emphasize the following:

*“Integrate risks and opportunities associated with climate change and energy management into the business strategy”* (ESG company report, 2020).

*“Proactively integrate climate change adaptation measures to increase the resilience of business and communities in the face of climate change impacts”* (ESG company report 2021).

*“Our decarbonization strategy is moving us towards a sustainable future by reducing fossil fuel-based energy consumption and related costs”* (ESG company report 2023).

## Discussion

This paper broadens the discussions relating to the feasibility of integrating environmental sustainability principles into the climate change adaptation measures in an effort to enhance the gold mine’s adaptive capacity and achievement of sustainable energy consumption.

### Impacts of Climate change on Energy use in Gold Mining

Climate change has emerged as a significant risk factor for the gold mining sector, particularly in South Africa’s Free State Province, where rising temperatures and extreme weather events are projected to intensify. Climate variability is both an environmental concern and a material business risk, directly influencing operational efficiency, worker safety, and energy consumption. Since 2020, the province has experienced an upward trend in average annual temperatures, with projections indicating that the frequency and severity of heatwaves will continue to rise. In certain areas, heatwave days may reach up to 50 annually, substantially heightening thermal stress on mining infrastructure and personnel.

Excessive heat is one of the most critical climate-related risks to gold mining operations, with implications for both underground and surface activities. All research participants (100%) acknowledged that rising temperatures have directly contributed to increased energy consumption, particularly for underground cooling and ventilation systems. Enhanced ventilation, improved compressed air systems, and optimized mine cooling technologies have become essential interventions to mitigate heat-related risks.

Despite these pressures, gold mining operations in the province have demonstrated a modest reduction in energy consumption. ESG reports indicate a gradual decline between 2020 and 2023, with a 3.6% reduction in electricity consumption recorded in 2023. This reduction is attributed to targeted energy efficiency and management programmes, reflecting organizational commitment to sustainability. However, these gains must be contextualized against escalating operational costs linked to climate change. Scholars such as Bartos (2007), Humphreys (2019), and Azadi et al. (2020) argue that rising energy demands and costs in South African mining are tied to climate impacts. Ebi et al. (2021) further note that global warming increases demand for cooling and ventilation, raising electricity expenses while extreme weather events such as floods and droughts cause infrastructure damage and operational disruptions.

Excessive heat and humidity also reduce equipment efficiency and increase maintenance costs. DESTEA (2024) underscores that higher temperatures negatively affect mineworker productivity due to heat stress and threaten infrastructure integrity. Declining ore grades necessitate more energy-intensive extraction processes, compounding the sector’s energy burden (Firoozi et al. 2024). The transition to a low-carbon economy adds complexity, as stricter carbon regulations pressure mining operations to adopt cleaner energy alternatives. Santaro et al. (2021) highlight that while these transitions are essential for sustainability, they often entail high upfront costs, particularly in retrofitting infrastructure. Taken together, these findings affirm that climate change drives energy use in the gold mining sector, necessitating comprehensive adaptation strategies. The successful implementation of energy efficiency projects in the Free State Province serves as a promising model, but continued innovation, investment in renewable energy, and integration of climate resilience into business strategies remain critical.

As Shemer et al. (2023) conclude, the cumulative effects of climate-related risks necessitate urgent, sustainable, and energy-efficient solutions to ensure the long-term viability of mining operations. This study reaffirms the need for adaptive responses that address both the direct and systemic impacts of climate change on energy use, worker safety, and operational sustainability in the gold mining industry.

### Environmental Sustainability Practices for Energy Conservation in Gold Mining

The mining industry, long associated with high energy consumption and environmental degradation, is increasingly adopting sustainability practices to address climate change and energy security. In South Africa, gold mining operations are progressively incorporating renewable energy and efficiency measures to conserve energy, reduce costs, and lessen reliance on fossil fuel-based power from Eskom. Several projects, including solar photovoltaic (PV) installations, wind energy wheeling, and small-scale hydropower, have been integrated into the energy mix alongside efficiency programs designed to lower electricity use and improve sustainability. All participants (100%) affirmed that energy-saving measures are being implemented, such as optimizing refrigeration units, upgrading ventilation systems with variable speed drives and inlet guide vanes, applying seasonal cooling controls, and load-shifting during peak periods. While the environmental rationale is widely acknowledged, 20% of respondents emphasized financial motivations, reflecting broader debates in sustainability literature. Energy efficiency contributes to conservation but also delivers immediate financial returns, particularly where Eskom’s power is carbon-intensive, costly, and unstable.

The integration of renewable energy serves multiple strategic objectives: mitigating GHG emissions, reducing fossil fuel dependency, and enhancing energy security. These actions align with findings from Farrant (2016), Mardani (2013), and Beg, Ali, and Rizwan (2002), who highlight the role of efficiency technologies in climate adaptation. They also resonate with Minerals Council South Africa (2022) and Votteler and Brent (2016), who note that gold mining operations pursue renewable energy to reduce carbon footprints, ensure supply reliability, and lower costs. Similarly, Aliyu, Modu, and Tan (2018), Uitto and Berg (2017), and Chicco (2010) emphasize renewable energy as both an environmental imperative and a cost-saving mechanism. Critics such as Zeghici and Polizu (2013) and Nelson and Schuchard (2011) argue that sustainability is constrained by historical dependence on fossil fuels. Nonetheless, Mytelka, Boyle, and Gorini (2016), Votteler and Brent (2016), and Dubiński (2013) present a more optimistic view, noting that South African mining companies are recognizing the unsustainable costs of fossil fuel reliance and prioritizing energy sustainability as a strategic objective.

In conclusion, while challenges remain, South Africa’s gold mining sector is making tangible progress in adopting sustainable energy practices. The dual goals of reducing carbon emissions and controlling energy costs are driving the adoption of renewable energy and efficiency measures. As climate pressures mount and energy costs rise, the integration of sustainability into mining operations is likely to accelerate, offering a pathway toward more responsible and resilient industry practices.

### The Interrelationship between Climate change and Energy use in Gold Mining

The growing body of evidence linking climate change to increased energy consumption is particularly relevant in energy-intensive industries such as gold mining. Findings from this study underscore a direct relationship between climate variability specifically the increase in heatwaves and extreme temperatures and energy usage in South African gold mining operations. This is most apparent in underground environments, where regulating heat exposure has led to greater reliance on ventilation and cooling systems. As heatwaves intensify, mines have extended the running hours of fans to maintain safe conditions, a measure essential for occupational health but one that significantly increases energy consumption and financial costs.

The Task Force on Climate-related Financial Disclosures [TCFD] (2021) report identified rising energy costs due to climate change as a key financial risk, highlighting the broader economic vulnerability of mining operations. These findings align with Müller (2018a) and Fattahi, Sadeghi, and Moradi (2018), who emphasize that energy costs are a major contributor to operational expenses as companies adopt more energy-intensive processes to cope with environmental challenges. Increased demand for climate control technologies compounds the sector’s environmental footprint when energy is fossil-fuel based. However, Rábago, Dworkin, and Smith (2001) argue that efficiency measures and innovative technologies can transform this necessity into economic opportunities, cutting costs while enhancing resilience. In this sense, energy conservation becomes both a business strategy and a climate mitigation approach.

Ebinger (2011) further contends that climate variability’s effects on energy-driven industries must be understood to ensure sustainability, as fluctuations in climate patterns directly influence demand, performance, and cost-efficiency. Overall, the connection between climate change and energy consumption in gold mining is multifaceted: rising temperatures compel increased energy use, driving up costs and risks, yet they also create impetus for innovation and sustainability. Future strategies must integrate climate resilience with energy efficiency planning to ensure adaptation measures contribute to operational safety, long-term economic viability, and environmental sustainability.

### Sustainability Principles, Adaptation Measures and Energy Optimization in Sustainable Mining

The study examined how environmental sustainability principles and climate change adaptation strategies intersect in gold mining operations, focusing on energy efficiency and renewable energy. A majority of participants reported that mining companies are applying sustainability principles, particularly sustainable energy consumption, to mitigate climate impacts and achieve sustainable mining. Energy efficiency emerged as a central pillar of both sustainability and adaptation, serving not only to reduce operational costs but also to enhance resilience against climate-related disruptions such as fluctuating energy prices, changes in energy availability, and extreme weather events.

Findings also highlighted the growing adoption of renewable energy sources, which align with broader sustainability goals by reducing fossil fuel dependence and strengthening operational resilience through more stable and cost-effective energy supplies. The integration of renewable energy into the energy mix functions simultaneously as a climate adaptation strategy and an energy optimization initiative, consistent with Rugină and Badea (2016), who emphasized the importance of renewable projects for sustainable development and adaptation. By lowering reliance on fossil fuels, mining operations reduce their carbon footprint and contribute to global climate mitigation efforts.

These results resonate with previous research (Farrant 2016; Rugină and Badea 2016; Davidson, Williamson and Foster 2003), which identifies synergies between adaptation and sustainable development goals. Energy-efficient technologies and renewable energy sources are both cost-effective and environmentally responsible, supporting sustainable operational practices while addressing climate challenges. This study affirms the views of Elum and Momodu (2017b) and Ley (2017b), who argue that renewable energy projects are integral to sustainable development and adaptation.

**Fig. 7:**
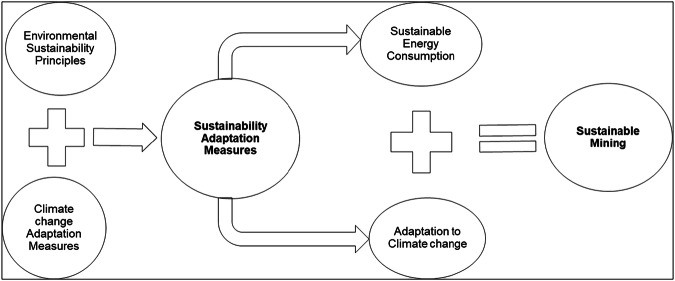
Conceptual Framework to Integrate Environmental Sustainability Principles and Climate Change Adaptation Measures to achieve Sustainable Mining (Source: Field data 2020 and 2021)

In conceptualizing “sustainability adaptation measures,” the study demonstrates that integrating energy efficiency and renewable energy strategies reduces consumption and emissions while strengthening adaptive capacity. This framework underscores the importance of embedding sustainability principles into mining business strategies to ensure long-term resilience. As illustrated in Fig. 7, sustainability adaptation measures,such as energy efficiency and renewable energy adoption are central to achieving both environmental and operational resilience in gold mining.

These sustainability adaptation measures are implemented to achieve sustainable energy consumption and adaptation to the impacts of climate change. Therefore, through the adoption of this pathway, it is understood that gold mining operations can achieve a sustainable mining business.

## Conclusion and Recommendation

This study demonstrates that climate change is significantly reshaping energy consumption patterns in South Africa’s gold mining sector, particularly in the Free State Province. Rising temperatures and climate variability are increasing the need for energy-intensive adaptation measures, such as enhanced ventilation and cooling. In response, gold mining operations are increasingly adopting energy efficiency technologies and integrating renewable energy sources to reduce operational costs, lower carbon emissions, and enhance resilience. The findings affirm a strong interconnection between environmental sustainability principles and climate change adaptation strategies. The implementation of energy-saving initiatives and renewable energy projects serves as a dual-purpose approach optimizing energy use while building long-term climate resilience. These sustainability adaptation measures are essential for ensuring the continued viability of gold mining operations in a changing climate.

This study further draws recommendations for mining companies to consider integrating environmental sustainability principles into climate adaptation planning particularly in the company strategies and policies to holistically address energy security and climate risks; invest in renewable energy and energy-efficient technologies to reduce dependence on fossil fuels and enhance operational resilience; conduct feasibility studies to identify cost-effective and context-appropriate adaptation strategies tailored to site-specific risks and; develop proactive energy and climate strategies that align with national sustainability goals and global best practices. By embedding sustainability into their adaptation frameworks, gold mining operations can position themselves as leaders in responsible mining and ensure long-term environmental and economic performance in the face of escalating climate challenges.

## Data Availability

This study utilized existing operational data from gold mining activities, interviews with company employees, and site visit observations. To protect participant privacy and company confidentiality, raw data and direct identifiers cannot be disclosed. However, aggregated and anonymized qualitative datasets are available. These include: Qualitative codes manually constructed from interview and questionnaire responses; Excel-based datasets containing coded narratives linked to events, with frequency counts of themes; Descriptive statistics (numbers and percentages) derived from frequency distributions of identified themes. The anonymized datasets and coding framework can be shared upon reasonable request to support reproducibility. No proprietary company data or identifiable participant information will be released.
